# Large cell neuroendocrine carcinoma of the nasal cavity: an extremely rare and new distinct entity

**DOI:** 10.11604/pamj.2018.30.188.14992

**Published:** 2018-06-29

**Authors:** Jawad Lahma, Reda Hejjouji, Philippe Gicquel, Leila Essakalli

**Affiliations:** 1 ENT Department, Ibn Sina University Hospital, Mohammed V University, Rabat, Morocco; 2 ENT Department, Hospital Center of Niort, France

**Keywords:** Large cell neuroendocrine carcinoma, nasal cavity, endoscopic endonasal surgery

## Abstract

Large cell neuroendocrine carcinoma (LCNEC) is a rare but distinct entity of nasal cavity carcinomas. Only a very few cases have been reported in the nasal cavity. Its treatment is not well established. We report the case of a LCNEC in a 70 years old patient who presented with right nasal obstruction and epistaxis since 2 months. Diagnosis was confirmed by histological and immunohistochemical study. The patient underwent complete endoscopic removal of the tumor combined to adjuvant chemoradiotherapy. After 6 month follow-up, the patient was in complete clinical and radiological remission. We will discuss in this paper the various epidemiology, clinical features, pathological findings, differential diagnosis, and evolution of treatment of this uncommon malignancy in the light of current knowledge. Optimal treatment strategies are yet to be determined for this rare malignancy with poor prognosis including surgery and chemoradiotherapy.

## Introduction

Neuroendocrine carcinoma is relatively uncommon in the sinonasal region. Very few cases of sinonasal large cell neuroendocrine carcinoma have been reported till date. In recent years, this variant has been recognized as a distinct entity. Otherwise, no agreement for adequate management has been reached. These aggressive tumors are requiring multimodality treatment that includes surgery as initial treatement and chemoradiotherapy. The focus of this paper is to report such extremely rare case and highlight recent developments in this pathology.

## Patient and observation

**Figure 1:**
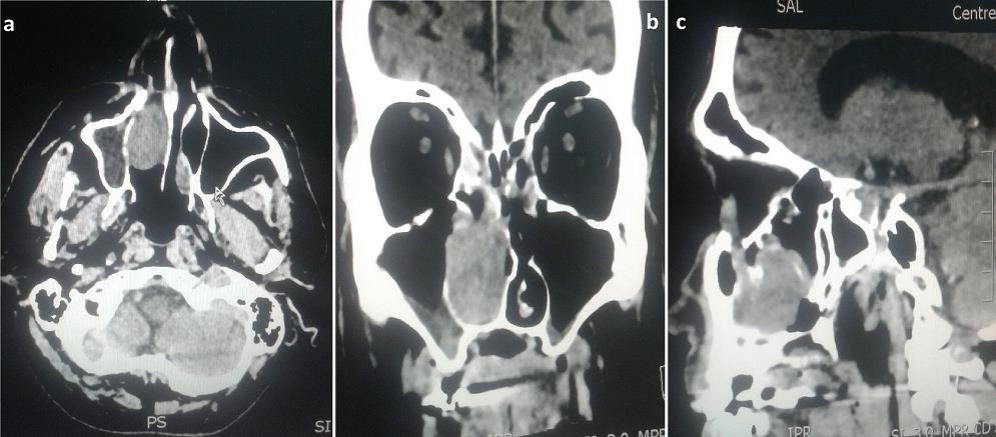
Sinonasal computed tomography (CT) viewed at soft tissue windows shows a right nasal mass filling the right-side nasal cavity enhanced after injection of contrast product: (A) axial post-contrast CT; (B) coronal post-contrast; (C) sagittal post-contrast CT

**Figure 2:**
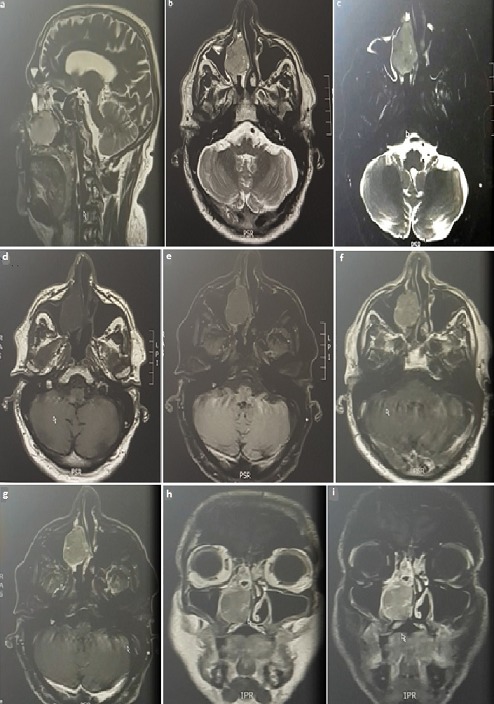
Nasosinusal MRI showing the tissue mass measuring 4 × 2.8 × 3.5cm with ethmoidal sinus fluid retention without intracranial or orbital extension (on sequence: (A) sagittal T2; (B) axial T2 TSE Fast; (C) axial T2 DIXON Fat; (D) axial T1 TSE dixon in; (E) axial T1 TSE dixon W; (F) axial T1 TSE dixon Gadolinium in; (G) axial T1 TSE dixon Gadolinium W; (H) coronal T1 Gadolinium; (I) coronal T1 Gadolinium W)

**Figure 3:**
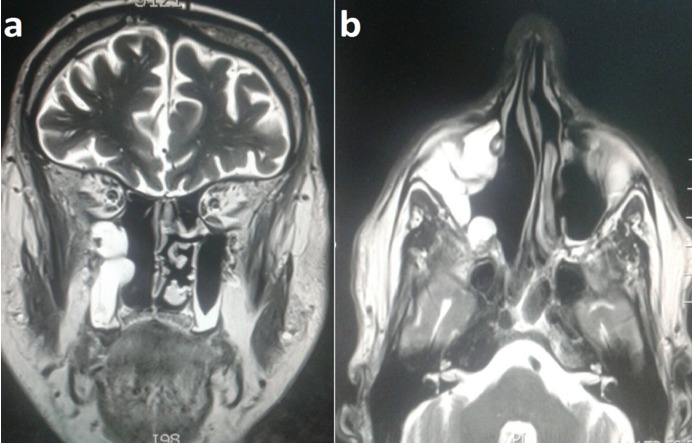
The post-treatment magnetic resonance imaging showed retention in the right maxillary sinus without suspicious mass detected

Mr F.B a 70-year-old male patient presented in our institution because of the occurrence of a right epistaxis. First of all, the patient is not smoking and reported an aneurysm of the abdominal aorta surgically treated by prosthesis under plavix. The beginning of the symptomatology goes back to 2 months before with right nasal obstruction of progressive onset, associated with right epistaxis and bloody rhinorrhea with right chronic eye watering. Nasal Endoscopy examination revealed a pinnkish burgeoning mass bleeding on contact and filling the right nostril vestibule. The patient didn't presents neurological signs nor cervical lymph nods. Otherwise, ophthalmologic examination found normal visual acuity and ocular mobility on both sides. Sinonasal computed tomography (CT) found a right nasal process enhanced after injection of contrast product with relatively little washing at the late time. Otherwise, there is no extension involvement at the level of the cavum and the pterygo palate fossa. This finding found also a crenate appearance of the posterior nasal mucosa especially of the middle turbinates and contralateral inferior turbinate with a left deviation of the nasal septum with nasal spur (Figure 1). Nasosinusal MRI shows a progressive increase in size of the tissue mass compared to the previously CT occupying the right nasal fossa on almost all of its height, measuring 4 × 2.8 × 3.5cm without extension within the cavum with probable invasion of the lower part of the nasolacrimal duct and mass effect on both the sinusonasal bone septum and the intersinusonasal septum. This finding shows ethmoidal sinus fluid retention. The appearance, even if it remains unspecific, is compatible with inverted papilloma (Figure 2). The patient underwent complete endoscopic removal of the tumor with ethmoidectomy and right maxillary antrostomy. The extemporaneous examination is in favor of a malignant tumor lesion. Histopathologic analysis shows a tumor proliferation made of polymorphic cells with major anisocytosis, most often located in lobules. There is sometimes a somewhat palisadic seat on the periphery of the tumor mass. The mitotic index is 10 mitoses per 10 high power fields. The immunohistochemical study of the tumor found that the cytokeratin KL1 and CK7 and the neuroendocrine marker Chromogranin A, synaptophysin and CD56 are positive. Proliferation index Ki67 is of the order of 80%. Otherwise there is an absence of expression of PS100, MelanA, HMB45, desmin, p40, CD45 and EBV. The clear positivity of cytokeratins formally excludes the diagnosis of olfactory neuroblastoma and leads to neuroendocrine carcinoma. Given the atypia, the mitotic index, and the Ki67 proliferation index, it is a high-grade large-cell neuroendocrine carcinoma. Extension assessment, comprising a PET SCAN, was negative. Postoperative treatment consisted in adjuvant polychemotherapy, with six cycles (cisplatin and etoposide), Followed by loco regional external radiotherapy. After 6 month follow-up, the patient was in complete clinical and radiological remission (Figure 3).

## Discussion

Neuroendocrine carcinomas (NECs) of the head and neck are a rare entity, regardless of differentiation or subtype, accounting for approximately 5% of sinonasal malignancies [1]. Larg Cell Neuroendocrine Carcinoma (LCNEC) is a high grade malignant neuroendocrine tumor that was first defined in the lungs by Travis et al. in 1991[2] and was not included as a diagnostic category; instead, it was noted that some moderately differentiated neuroendocrine carcinomas “may fulfill the diagnostic criteria of LCNEC of the lung” [3]. Despite this, in recent years LCNEC of the head and neck has been recognized as a distinct entity. Only a few cases of LCNEC have been reported in the sinonasal region and nasopharynx possibly because they are underreported or have been included under other non-descriptive categories [4]. Otherwise, Forthy-eight cases have been reported to date, in patients aged 9 to 88 years (mean, 61y). There is a strong male predilection, with 38 of 48 cases (75%) arising in men. The affected sites have been the larynx (n=25), the parotid gland (n=6), the oropharynx (n=6), the nasopharynx (n=3), the sinonasal tract (n=7), and the hypopharynx (n=1). Most patients have been smokers [3-20]. Clinical presentations of Sinonasal NECs include non-specific symptoms of nasal obstruction, epistaxis and/or facial pain. Extension into the nasopharynx, skull base, orbit, or brain may occur in poorly differentiated NECs with symptoms such as palpable facial mass and exophthalmia [21]. Paraneoplastic syndrome, particularly syndrome of inappropriate secretion of antidiuretic hormone (SIADH) has been reported [22, 23]. The most frequent sites for distant metastases are the lungs, liver, and bone [24]. Histoligically LCNEC is defined as tumor composed of organoid nests, trabeculae and rosettes, often with peripheral palisading of nuclei. The cells are large with relatively low nucleus/cytoplasm ratio, nucleoli or vesicular chromatin and a high mitotic rate >11 mitoses or greater per 2 mm^2^ or 10 high power fields. Although not used for grading or prognosis, Ki-67 labeling index is >20% [7]. Neuroendocrine features are seen on immunohistochemistry or electron microscopy. Radiological examination by combination of CT and MRI is important to determine the hallmarks of the local tumor invasion and are better for the planning of further treatment [24, 25]. The kadish staging system is used to describe the tumor extent [26]; kadish A: limited to nasal cavity, kadish B: limited to nasal cavity and paranasal sinuses, kadish C: tumor extending beyond the nasal cavity and paranasal sinuses.

Differential diagnoses include sinonasal undifferentiated carcinoma (SNUC), malignant melanoma, and lymphoma. Rarity of cases and overlapping morphologic and immunohistochemical features pose a conceptual challenge in the diagnosis of LCNEC and its differentiation from SNUC and high-grade olfactory neuroblastoma (ONB) [27]. Aggressive trimodality therapy seems to be the most effective approach, although survival remains poor. Surgery supplemented with postoperative radiotherapy or concomitant chemoradiotherapy is accepted as the primary treatment for localized disease. For patients with systemic disease, palliative chemotherapy or best supportive care remains appropriate options [27]. In a meta-analysis, van der Laan et al rapported that, irrespective of the histological diagnosis of NEC, surgery has a beneficial effect on survival and should be the cornerstone of any treatment strategy. This is supported by both the univariate and multivariate analyses in which treatment (combinations) incorporating surgery produced the best results. Otherwise Radiotherapy as monotherapy should not be performed in curative setting. No benefit from the application of chemotherapy could be deduced from his results. Chemotherapy as monotherapy had the worst 5-year median disease-specific survival, with no patients surviving regardless of tumor subtype [13]. LCNEC has an aggressive behavior, similar to that of small cell carcinoma and a poorer prognosis when compared to atypical carcinoid tumors; 5 year survival rates of atypical carcinoid tumor, LCNEC, and SCNEC were 83.3, 21.4, and 20.8%, respectively in a study of 23 NEC, 11 of which occurred in the sinonasal tract [7]. In a review of 10 laryngeal LCNECs by Lewis et al. [8], 9 patients (90%) presented with stage IV disease and 60% died of their disease. Kasafuka et al. [9] in a series of 8 mucosal LCNEC reported that 7 of 8 (80%) patients presented with regional lymph node metastases and 3 had died of disease after follow-up ranging from 15 to 90 months. Three of 4 (75%) patients with well documented LCNEC of the salivary glands have died of their disease [16-27]. In van der Laan et al meta-analysis, most patients with laryngeal LCNEC presented with advanced disease and that the 5-year disease-free survival was 15%. By comparison, the 5-year survival rates of laryngeal small cell carcinoma and moderately differentiated euroendocrine carcinoma were 19% and 53%, respectively [13].

## Conclusion

Large cell neuroendocrine carcinoma of the nasal cavity is an extremely rare and destructive neoplasm. Clinical presentations of this uncommon malignancy include non-specific symptoms. Aggressive trimodality therapy seems to be the most effective approach to improve locoregional control and survival.

## Competing interests

The authors declare no competing interests.
